# A Fine Grain, High Mn Steel with Excellent Cryogenic Temperature Properties and Corresponding Constitutive Behaviour

**DOI:** 10.3390/ma11020253

**Published:** 2018-02-07

**Authors:** Yuhui Wang, Baodong Shi, Yanming He, Hongwang Zhang, Yan Peng, Tiansheng Wang

**Affiliations:** 1 State Key Laboratory of Metastable Materials Science and Technology, Yanshan University, Qinhuangdao 066004, China; yhwang@ysu.edu.cn (Y.W.); ymhe@stumail.ysu.edu.cn (Y.H.); 2 National Engineering Research Center for Equipment and Technology of Cold Strip Rolling, Yanshan University, Qinhuangdao 066004, China; baodong.shi@ysu.edu.cn (B.S.); hwzhang@ysu.edu.cn (H.Z.); pengyan@ysu.edu.cn (Y.P.)

**Keywords:** high Mn steel, cryogenic temperature properties, product strength and uniform elongation, constitutive behaviour

## Abstract

A Fe-34.5 wt % Mn-0.04 wt % C ultra-high Mn steel with a fully recrystallised fine-grained structure was produced by cold rolling and subsequent annealing. The steel exhibited excellent cryogenic temperature properties with enhanced work hardening rate, high tensile strength, and high uniform elongation. In order to capture the unique mechanical behaviour, a constitutive model within finite strain plasticity framework based on Hill-type yield function was established with standard Armstrong-Frederick type isotropic hardening. In particular, the evolution of isotropic hardening was determined by the content of martensite; thus, a relationship between model parameters and martensite content is built explicitly.

## 1. Introduction

The development of materials with higher strength and improved plasticity is a never-ending pursuit of researchers [1,2,3,4]. The product of ultimate tensile strength and uniform elongation (PSE) are quite important performance indices in material design. A PSE exceeding 30 GPa, proposed by the third-generation automobile steels [5], and a high PSE value display constitute a huge challenge under low temperatures due to brittle fracture at cryogenic temperatures [6].

High manganese austenitic steels have been widely studied for their corresponding outstanding tensile strength and ductility [7,8,9,10]. Certain high Mn austenitic steels have also been investigated for cryogenic applications [11,12,13,14]. Recently, Koyama et al. [6] studied the effects of temperature on the tensile properties of Fe-17Mn-0.6C steels and discovered that the uniform tensile elongation deteriorated as the deformation temperature decreased from room temperature to −100 °C [15]. The lower ductility was attributed to an increase in the amount of ε-martensite plates. This suggestion was supported by the observation that an addition of elements such as carbon, which suppressed the formation of ε-martensite, improved the cryogenic tensile ductility [15,16]. Therefore, it has been suggested [16] that the reduction number of ε-martensite plates is a key factor for the cryogenic tensile ductility improvement of high Mn steels.

One way for the number of ε-martensite plates to be suppressed is through austenite grain size refinement [17,18]. Takaki et al. [17] investigated the grain size effects on ε-martensite formation in Fe-15Mn alloys and discovered that when the grain size was below 10 µm, the ε-martensite transformation was almost suppressed. In a quite recent study [10], it was demonstrated that Fe-17Mn-0.6C alloys with coarse grain sizes (10–37 µm) demonstrated a brittle fracture at −150 °C due to ε-martensite formation. In contrast, this embrittlement was suppressed by grain refinement to 3.5 µm. The formation of ε-martensite could also be suppressed by an increase in the Mn content. Tomota et al. [8] demonstrated that, regarding the binary Fe-Mn system, when the Mn content was increased to 36%, no ε-martensite was observed in the tensile tested samples at room temperature, whereas only 4.2% of ε-martensite was formed following tensile deformation to a strain value exceeding 60% at the liquid nitrogen temperature.

Fe-34.5Mn-0.04C steels are recently developed steels with potential applications at low temperatures [19]. In this paper, a Fe-34.5Mn-0.04C alloy was prepared for the corresponding mechanical behaviour study as a function of temperature within the range of room temperature to −150 °C, with an aim to determine the temperature range in which the steel demonstrated an excellent combination of tensile strength and ductility. Moreover, the constitutive behaviour of high manganese austenitic steels was fully characterised.

## 2. Experimental Procedure

The steel utilised in this study, had a nominal composition of Fe-34.5Mn-0.04C. An ingot was produced from a vacuum induction furnace and subsequently forged in the temperature range of 800–1100 °C to form a 13-mm thick plate. The plate was subsequently cold rolled to a thickness reduction of 90% by a laboratory rolling mill with a roll diameter of 230 mm, rotated at 35 r/min. The recrystallisation annealing treatments were performed in a vacuum furnace at 800 °C for 1 h. The tensile specimens with the gauge dimensions of 50 mm in length, 12.5 mm in width, and 1.3 mm in thickness were machined from the recrystallised sheets. The tensile tests were executed at a strain rate of 10^−3^·s^−1^ at various temperatures from −150 °C to room temperature (RT), using the method of spraying liquid nitrogen to obtain different temperatures. A thermocouple was used to measure the temperature. After reaching the set temperature, it was held for 5 min, and then the tensile test was started. A FEI-Scios DualBeam™ system (FEI, Brno, Czech Republic) operated at 15 kV was used for microstructural investigation. Electron backscatter diffraction (EBSD) (EDAX, Mahwah, NJ, USA) measurements were carried out on the plane parallel to the rolling and normal direction (RD-ND); scans with a step size of 0.1 µm were carried out using a TSL-OIM EBSD attachment (EDAX, Mahwah, NJ, USA), and the specimen surface was mechanically polished followed by electrochemical polishing in a 2:1:7 HClO_4_:C_3_H_8_O_3_:C_2_H_5_O solution at 0 °C for 60 s. The microstructural examinations were performed along the rolling and transverse direction (RD-TD) plane by X-ray diffraction (XRD) (Rigaku Corporation, Tokyo, Japan). The XRD data acquisition was executed by Cu Kα radiation by a powder diffractometer. The step scan mode was employed, with a preset holding duration of 2 s at each 0.02° step in a 2θ from 40° to 104°. Specimens for TEM (FEI, Hillsboro, OR, USA) investigations were cut parallel to the RD/ND plane (longitudinal section) and TEM foils were prepared by a twin-jet technique in perchloric-based electrolytes.

## 3. Results and Discussion

### 3.1. Tensile Properties of Investigated Steel at Various Temperatures

**Figure 1:**
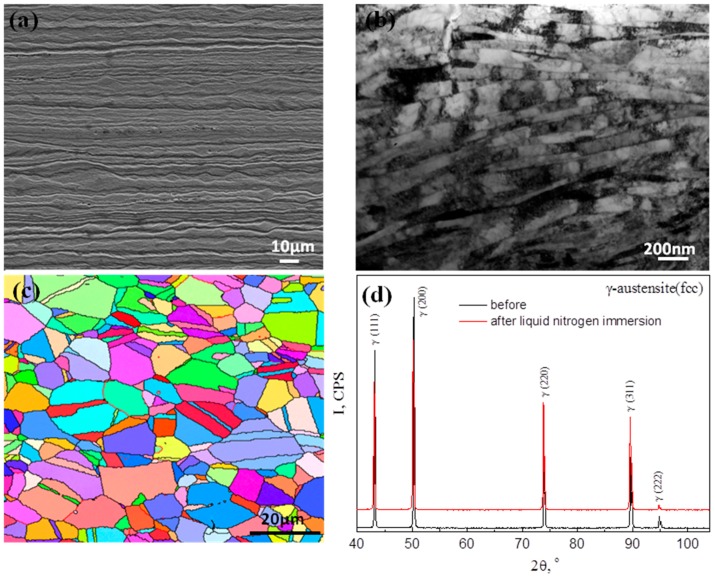
(**a**) SEM image and (**b**) TEM image showing the deformed microstructure of cold-rolled microstructure, Electron back-scattered diffraction (EBSD) orientation map; (**c**) recrystallised fine grain structure of the material that was cold rolled and annealed for 1 h at 800 °C; (**d**) X-ray diffractograms of samples prior to and following liquid nitrogen immersion for 20 min.

Figure 1a presents micrographs of the investigated steel following the final cold rolling 90% reduction. A banded structure with the band direction parallel to the rolling direction is seen from the optical image (Figure 1a). The band widths vary from about 1 µm to about 10 µm. Some of the bands show somewhat wavy features that are associated with localised shear deformation [20,21]. TEM observation (Figure 1b) shows the formation of a fine scale deformed lamellar structure, with the lamellar boundaries approximately parallel to the RD. Figure 1c shows the recrystallised grain structure where the mean grain size was measured to be 3.8 µm, with the annealing twin boundaries also taken into consideration for the grain size measurements by EBSD. The same grain size of 3.8 μm, obtained in the previous study [19], was reproduced after annealing at 800 °C for 1 h. Brass and Cube texture components were observed in the alloy. However, the texture intensities were relatively low. The textural evolution during the tensile test could be neglected since the deformation strains were small. The X-ray diffraction confirmed that the recrystallised grain structure was a stable full austenite, both prior to and following liquid nitrogen immersion for 20 min (Figure 1d).

**Figure 2:**
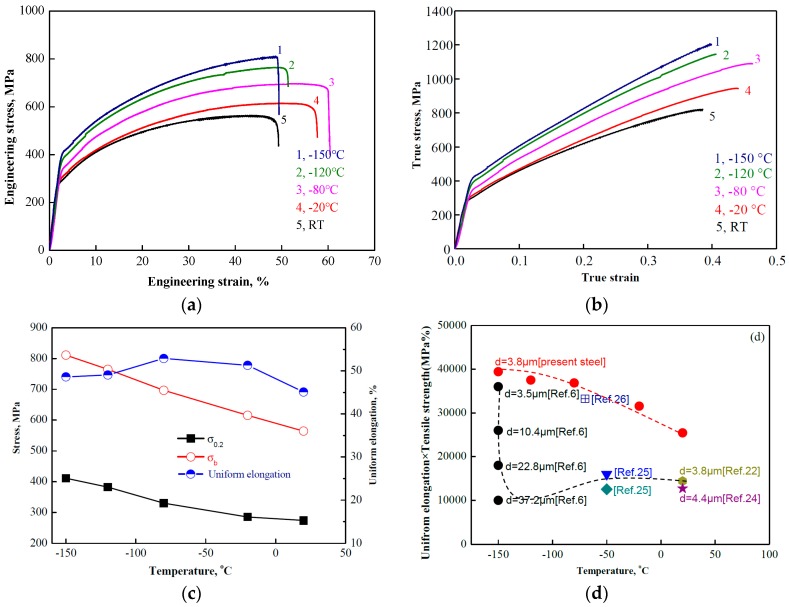
Stress-strain curves of investigated steel, tested at various temperatures. (**a**) Engineering stress-engineering strain; (**b**) True stress-true strain; (**c**) Temperature dependence of tensile properties; (**d**) temperature and PSE (the product of ultimate tensile strength and uniform elongation) relationship.

Figure 2a presents the stress-strain curves of the investigated steel tested at RT, −20, −80, −120, and −150 °C. At RT, the yield strength (σ0.2), the ultimate tensile strength (σb), and the uniform elongation were 274 MPa, 564 MPa, and 45%, respectively. These tensile properties were slightly higher than the reported tensile properties of binary Fe-(30–36) Mn alloys [8,22,23], probably due to the difference in Mn contents and to the contribution of low amounts of carbon in the present alloy. As the temperature decreased, the yield strength gradually increased (Figure 2a), reaching 382 MPa at the lowest testing temperature of −150 °C. The work hardening rate was enhanced significantly as the temperature decreased, as it could be observed from the slope change of the true stress-true strain curve, shown in Figure 2b. The ultimate tensile strength displayed a continuous increase as the temperature decreased. The uniform elongation was approximately 45–53% over a wide range of temperatures from RT to −150 °C (Figure 2c).

Figure 2d displays the PSE of various high manganese steels at room and cryogenic temperature. At room temperature, the tensile strength of the investigated steel significantly exceeded that of the Fe-33Mn [22] steel with a grain size of 3.8 µm and that of the Fe-24Mn-3Al-2Si-1Ni-0.06C [24] steel with a grain size of 4.4 µm. Regarding the low temperature performance in comparison that reported by other scholars, the investigated steel PSE was significantly better than the Fe-28Mn-1.6Al [25], the Fe-27Mn-4.1Al [25], and the Fe-30Mn-3Al-3Si [26] at the temperature test range of −40 °C to −80 °C. The present steel PSE with a grain size of 3.8 µm (39,433 MPa%) was increased by 9.5% compared to that of the Fe-17Mn-0.6C [10] steel with the grain size of 3.5 µm (PSE = 36,000 MPa%), and was increased by 294% compared to the steel with a grain size of 37.2 µm.

Among all fully recrystallised, high Mn steels, it was discovered that the present steel display of a fine grain size exhibited the highest PSE exceeding 30 GPa in the temperature range of −20 °C to −150 °C. This was the first report demonstrating a fully recrystallised, fine-grained Fe-34.5Mn-0.04C steel with excellent low temperature properties.

**Figure 3:**
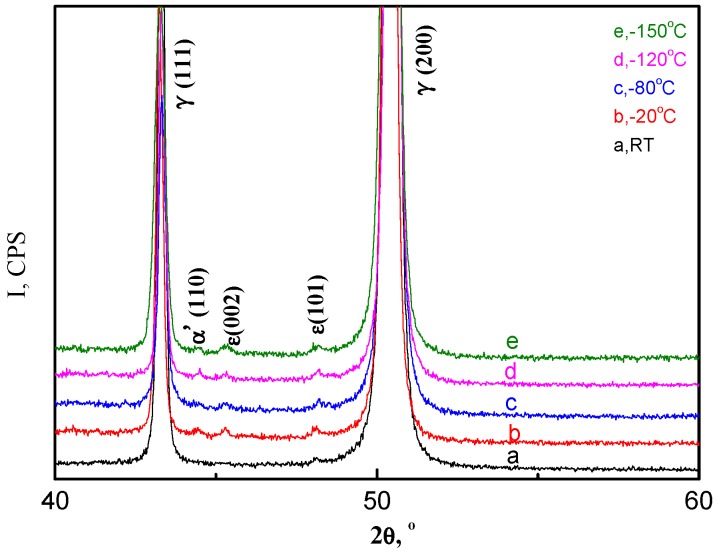
X-ray diffractograms of various test temperatures: RT, −20 °C, −80 °C, −120 °C, and −150 °C.

Whether this mechanism applied to the present alloy or not, X-ray diffraction was executed for all tensile-tested samples for the phases present in the specimens, and the phase quantification of both austenite and martensite were determined by the Rietveld method (Table 1). The Rietveld full spectrum fitting method was employed. The five examples of X-ray diffraction results obtained from the samples tested at RT, −20, −80, −120, and −150 °C are presented in Figure 3. Following RT deformation (48% elongation), the samples still displayed a fully austenitic structure. Following testing at −20 °C (58% elongation), low-sized diffraction peaks corresponding to the α’-martensite and ε-martensite appeared, indicating that a low amount of α’-martensite and ε-martensite was formed during tensile deformation. Subsequent to testing at −80 °C (60% elongation), an approximately 3.1% α’-martensite and ε-martensite formation occurred, whereas following testing at −150 °C, a 5.7% α’-martensite and ε-martensite formation occurred. The higher strength and ductility was attributed to the number of martensite plates suppressed by the combined aspects of grain refinement and higher Mn content in our study.

### 3.2. Constitutive Description of Mechanical Behaviour at Various Temperatures

Based on the aforementioned microstructure description, the material transformation at various temperatures could be partially understood. In contrast, must be mentioned that the underlying structure evolution could not be fully characterised without a clear understanding of the constitutive relations. Consequently, in this section, a common Hill-type yield function combined with the Armstrong-Frederick-style isotropic hardening was employed within the finite strain plasticity framework [27,28,29,30,31,32,33]. The deformation gradient F=GRADφ was multiplicatively decomposed into an elastic part *F^e^* and a plastic part *F^P^*. Analytically:

(1) $$F=F^{e}\cdot F^{P},\;\mathrm{with}\;\det F^{e}>0,\;\det F^{P}>0$$

Within a thermodynamic framework, the constitutive response of the high manganese austenitic steels could be described by a Helmholtz energy Ψ. In line with the underlying assumptions of the kinematics in Equation (1), the Helmholtz energy Ψ was also uncoupled into an elastic part Ψ*^e^* (due to the elastic response of crystals) and a plastic part Ψ*^P^* (due to plastic work and hardening), such as:

(2) $$\Psi =\Psi^{e}\left(F^{e}\right)+\Psi^{P}\left(\alpha \right)$$

In the current work, a stain-like internal variable α for the isotropic hardening was introduced in the Ψ*^P^*. Based on the standard Coleman and Noll procedure [28], the first Piola Kirchhoff stress is:

(3) $$P=\frac{\partial \Psi }{\partial F}$$

and the reduced dissipation inequality is:

(4) $$D=\Sigma :L^{P}+Q:\dot{\alpha }\geq 0,\;Q:=-\frac{\partial \Psi }{\partial \alpha }$$

where $\sum =2C^{e}\cdot \frac{\partial \Psi }{\partial C^{e}}$ is the Mandel stress tensor, L*^P^* is the plastic velocity gradient, and *Q* is the stress-like internal variable work conjugated to α. The model was closed by a space definition of admissible stresses $E_{\Sigma }$. According to the reduced dissipation Equation (4), this space is described as:

(5) $$E_{\Sigma }=\left\{\left(\Sigma ,Q\right)\in {\mathbb{R}}^{9+n}|\phi \left(\Sigma ,Q\right)\leq 0\right\}$$

In line with Equation (5), the admissible space $E_{\Sigma }$ is constrained by the yield function *φ*. Usually, the yield function is assumed to be convex. The loading and unloading conditions (Karush-Kuhn-Tucker conditions) are defined by:

(6) $$\lambda \phi =0,\lambda \dot{\phi }=0$$

where λ > 0 is the plastic multiplier computed from the consistency condition $\dot{\phi }=0$ with plastic deformation. With a focus on the high manganese austenitic steels, a common Hill-type yield function is adopted [34], such as:

(7) $$\phi :={\left[\left(dev\Sigma -Q_{kin}\right):ℍ:\left(dev\Sigma -Q_{kin}\right)\right]}^{\frac{1}{2}}-Q_{iso}-Q_{0}$$

**Figure 4:**
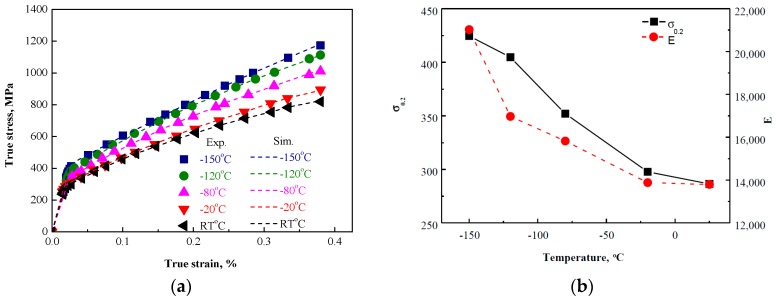

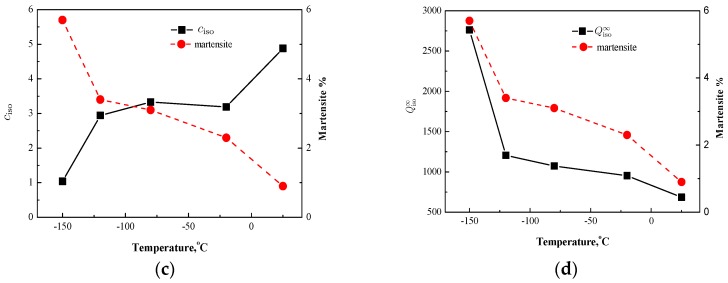
Comparison between experimental observations and numerical simulations (**a**); initial yield stress evolution and Young’s modulus versus temperature (**b**); model parameter *C_iso_* evolution and martensite content versus temperature (**c**); model parameter $Q_{iso}^{\infty }$ evolution and martensite content versus temperature (**d**).

As the anisotropic mechanical behaviour was not focused on in the current work, only the isotropic hardening effect was considered. Therefore, a simplified yield function was applied instead, quite explicitly as:

(8) $$\phi :={\left(dev\Sigma :{ℍ}_{0}:dev\Sigma \right)}^{\frac{1}{2}}-Q_{iso}-Q_{0}$$

where ℍ_0_ is the fourth order deviatoric identity tensor, *Q_iso_* is a stress-like internal variable denoting the isotropic hardening, and *Q*_0_ is the initial size of the yield surface. Based on the Hill-type yield function employed in the current work, *Q*_0_ can be obtained by a uniaxial tensile test without the strength differential effect. Therefore, *Q*_0_ is the same value as σ_0.2_ by experimental observations. (Because the flow is continuous and the yield point is not observed in the systems, *Q*_0.2_ could be utilised as *Q*_0_). Concerning the yield surface evolution, a typical Armstrong-Frederick-style hardening law is employed, such as:

(9) $${\dot{Q}}_{iso}=\lambda C_{iso}\left(Q_{iso}^{\infty }-Q_{iso}\right)$$

where *C_iso_* and $Q_{iso}^{\infty }$ are model parameters characterising the saturation rate and value of *Q_iso_*, respectively. Regarding the high Mn steel discussed in the current work, the plastic deformation, such as the lower true strain below 0.40, was focused on. Based on the least squares method, the model parameters were calibrated, whereas the comparison between the experimental observations and numerical simulations are presented in Figure 4a. The model parameters evolution and the martensite content are demonstrated in Figure 4b,c.

Firstly, the Young modulus increased as the temperature decreased, as presented in Figure 4b. According to Reference [33], it was believed that the Young modulus was affected by the crystal lattices’ elastic distortion and the binding force between atoms. The deformation temperature decrease led to a decrease in the atomic spacing, whereas, consequently, the binding force between atoms increased and the Young modulus increased. As the yield surface initial size, *Q*_0.2_ characterised the yield stress. As the deformation temperature decreased, *Q*_0.2_ increased, which occurred due to the microstructural transformation (Figure 4a). With a focus on the plastic deformation, the evolution of *C_iso_* determined the saturation rate of *Q_iso_*, along with the evolution rate of stress [30,31,35]. Based on Figure 4c, it was clear that a counteraction effect between the martensite content and the evolution rate of stress existed, as the martensite content increased as the temperature decreased, whereas the *C_iso_* decreased. Naturally, as the temperature decreased, the martensite content increased, which led to the material strengthening effect, requiring a higher external force for deformation. This was consistent with the linear relation between *C_iso_* and *M* (the martensite content):

(10) $$C_{iso}\approx 5.425-76.236\times M$$

With a focus on $Q_{iso}^{\infty }$, the relationship between $Q_{iso}^{\infty }$ and *M* was clear (Figure 4d), whereas at temperatures exceeding −120 °C:

(11) $$Q_{iso}^{\infty }\approx 504.86+195.67\times M$$

This could be interpreted as, with the decrease in temperature, the martensite content increased, whereas the *Q_iso_* saturation level along with the saturation stress increased. Naturally, the martensite content increase led to the stress limit level increase with a simple linear approximation. Regarding the temperature decrease below −150 °C, a strong increase of $Q_{iso}^{\infty }$ was observed, which was quite higher than the expected value in line with Equation (11). This occurred due to strong microstructural changes and could result in a drastic change in mechanical behaviour.

## 4. Conclusions

In summary, a fully recrystallised fine-grained structure of the investigated steel was obtained through a conventional cold rolled 90% reduction and annealing at 800 °C for 1 h. The steels exhibited excellent low temperature properties in the temperature range of −20 °C to −150 °C: a uniform elongation exceeding 48.6%, a tensile strength increase from 615.3 MPa to 811.4 MPa, and a PSE exceeding 30 GPa. The mechanical behaviour was fully characterised by the constitutive model with Armstrong Frederick (AF) isotropic hardening. In particular, the evolution of isotropic hardening was governed by the microstructure, and two linear relationships between model parameters for isotropic hardening and martensite content were obtained.

## Figures and Tables

**Table 1:** Phase quantification of austenite and martensite by the Rietveld method.

Tensile Temperature	Room Temperature	−20 °C	−80 °C	−120 °C	−150 °C
Martensite	-	2.3%	3.1%	3.4%	5.7%
Austenite	100%	97.7%	96.9%	96.6%	94.3%
